# Exploiting Marker Genes for Reliable Botanical Authentication of *Bacopa monnieri* Products

**DOI:** 10.3390/foods14183275

**Published:** 2025-09-21

**Authors:** Rita Biltes, Caterina Villa, Joana Costa, Isabel Mafra

**Affiliations:** REQUIMTE/LAQV, Faculdade de Farmácia, Universidade do Porto, Rua de Jorge Viterbo Ferreira, 228, 4050-313 Porto, Portugal; rbiltes@ff.up.pt (R.B.); cvilla@ff.up.pt (C.V.); jbcosta@ff.up.pt (J.C.)

**Keywords:** *Bacopa monnieri*, Brahmi, real-time PCR, authentication, botanical adulteration, quantification

## Abstract

*Bacopa monnieri*, commonly known as Brahmi, is a perennial herbaceous plant used in Ayurvedic medicine owing to its nootropic properties. The increased demand for bacopa-derived herbal/food products has motivated adulteration practices through plant substitution. This work is aimed at developing a new method for *B. monnieri* detection and quantification in herbal products. The chloroplast gene encoding the Ycf1 photosystem I assembly protein (*Ycf1*) and the nuclear gene coding for the flavonoid glucosyltransferase (Flag) were selected as candidate markers to develop a real-time PCR assay with EvaGreen dye for *B. monnieri* detection. Both markers were specific to the target species, with *Ycf1* providing the best real-time PCR kinetics and highest sensitivity. Therefore, a new method targeting the *Ycf1* barcode was developed, exhibiting high specificity and a sensitivity of 1 pg of bacopa DNA. Additionally, a calibration model was proposed using reference mixtures of *B. monnieri* in *Ginkgo biloba* with a linear dynamic range of 25–0.1% (*w*/*w*). The curve parameters of slope, PCR efficiency and correlation coefficient met the acceptance criteria. The method was successfully validated with blind mixtures and further applied to commercial herbal products, revealing an important level of adulteration in bacopa/Brahmi-labelled products (60%) due to absence of or reduction in bacopa content. In this work, the first quantitative real-time PCR method for the botanical authentication of *B. monnieri* in herbal products is proposed as a powerful tool, which can be used by quality control laboratories and regulatory authorities to ensure labelling compliance.

## 1. Introduction

*Bacopa monnieri* (L.) Pennell, commonly known as Brahmi, hyssop, herb of grace or water hyssop, is a small, non-aromatic perennial herbaceous plant belonging to the Plantaginaceae family [1]. It is commonly found growing in South and Southeast Asia, Africa, America and Australia, typically in warm marsh habitats [2]. *B. monnieri* has a long history in traditional medicine, being used for thousands of years as a therapeutic herb in Ayurvedic medicine in India, mainly as a nerve tonic and nootropic booster [1]. Additionally, it has been reported to have other pharmacological actions such as antioxidant, anticancer, antidiabetic, analgesic, hepatoprotective, cardiotonic and diuretic activity [2,3]. *B. monnieri* contains bioactive compounds such as alkaloids, saponins, glycosides, flavonoids, triterpenes and cucurbitacin [4]. Specifically, some examples are brahmine, bacosides A and B, saponins A, B, and C, aspartic acid, brahmic acid, betulinic acid, beta-sitosterol, apigenin and serine [5], of which bacoside A and betulinic acid are believed to have the main therapeutic action [5,6].

*B. monnieri* can be used as a whole plant or an extract, on its own or in combination with other nootropic herbs in various dosage forms, including as a fresh juice, soft paste of fresh herbs, product processed with clarified butter, oil or fine dry powder or tablet [7]. Additionally, novel product formulations have been tested to incorporate *B. monnieri* plant or its extracts into different food products such as candy, cereals, dried food products, soup mixes, herbal whey beverages and meat products, some being already available in the Indian market [8,9,10]. The primary cultivation countries of *B. monnieri* are India, Nepal and Sri Lanka [11]. The market is dominated by the Asian Pacific region (33%), followed by North America (25%), and the “Brahmi market” is forecasted to reach $320 million by 2026, with a compound annual growth rate (CAGR) of 6.1% during 2021 to 2026 [12]. Corroborating this data forecast, in 2022, the global market for brain health supplements, including *B. monnieri* health products, was valued at $8.2 billion and is expected to nearly double by 2030, with a CAGR of 8.3% [13,14]. The expansion of the market size suggests a growing demand for herbal products, which may lead to an increased risk of adulteration, involving unintended or deliberate plant swaps, contamination or addition of illegal substances or admixtures [15]. In India, *B. monnieri* and *Centella asiatica* are often sold under the same common name – Brahmi – leading to confusion between the two species [16]. Moreover, *B. monnieri* is by itself a common adulterant of *Portulaca oleracea* due to the morphological similarities of both plants [17,18].

Health products containing botanicals such as *B. monnieri* primarily fall under the category of food supplements, thus being regulated as foods by the European Food Safety Authority and not requiring any approval before commercialisation by the Food and Drug Administration (FDA) [15]. Additionally, the European Union (EU) novel catalogue [19] does not classify *B. monnieri* as a novel food, since it has been used as such before 15 May 1997. Therefore, it is not subject to pre-market authorisation according to Regulation (EU) 2015/2283. However, these products must comply with Regulation (EU) No 1169/2011 on the provision of food information to consumers and Directive 2004/24/EC regarding traditional herbal medicinal products [20]. On the other hand, the FDA has not approved products containing this plant for medical purposes. In fact, in 2019, warnings were issued to dietary supplement manufacturers who produce products containing *B. monnieri*, cautioning against making any therapeutic claims regarding its use [21]. Considering this, ensuring the safety, quality and trustworthiness of the *B. monnieri* health products is essential.

Therefore, the development of analytical methodologies for the botanical authentication of herbal products and plant food supplements are of utmost importance to ensure their safety and quality. Several methodologies have been currently proposed for this purpose, including morphological identification, chromatographic analysis of phytochemical compounds and genetic approaches based on DNA markers. DNA-based methods have been proposed as highly suitable for the botanical authentication of complex and processed matrices such as raw and processed botanicals, herbal products, and plant food supplements [22]. They provide highly specific, sensitive, robust and reliable tools targeting markers ubiquitous in all tissues, independent from the part of the plant, physiological conditions and environment, in opposition to phenotypic and chemical markers. Several DNA-based approaches have been successfully applied to authenticate herbal products, namely DNA barcoding, species-specific PCR, real-time PCR, sequence-characterised amplified region (SCAR) and high-resolution melting (HRM) analysis, among others [22,23]. Real-time PCR is the technique of choice of many laboratories because of its quantitative aptitude and applicability to highly processed food matrices.

To date, few studies using DNA-based methodologies have been conducted to authenticate *B. monnieri* plant or herbal commercial products. A randomly amplified polymorphic DNA (RAPD)-based SCAR method was proposed for the identification of *B. monnieri* from its potential adulterants, namely *Centella asiatica*, *Eclipta alba* and *Malva rotundifolia* [24]. Tungphatthong et al. [25] developed a HRM analysis targeting a barcode marker (*trnL-F*) to differentiate *B. monnieri* from *B. caroliniana* and *B. floribunda* species. Thakur et al. [26] exploited amplicon length polymorphisms using several universal DNA barcode regions, of which *atpF-atpH*, *trnH-psbA* and *trnL* allowed for discrimination of 46 medicinal plant species, including *B. monnieri* and *C. asiatica*. Shah et al. [16] aimed to develop SCAR marker-based PCR and metabarcoding methods to authenticate Brahmi herbal products, comparing them with chemical approaches. Results showed a relatively low success rate for SCAR marker PCR amplification, while the metabarcoding approach was able to detect the target and non-target species. Xu et al. [18] developed species-specific PCR and real-time PCR assays to detect *B. monnieri* as a potential adulterant of *Portulaca oleracea*. The qualitative PCR assay was successfully applied to analyse commercial herbal products, but without providing any quantitative determination.

This work aimed at developing a new real-time PCR method for *B. monnieri* detection and quantification in herbal products. For this purpose, several DNA sequences were in silico analysed to identify candidate marker sequences specific for the target species. The best marker was selected to propose a sensitive, specific and cost-effective tool. Accordingly, a calibration model was developed using model mixtures of *B. monnieri* in *Ginkgo biloba* leaf material. The choice of *G. biloba* was due to its most popular use for medicinal purposes, having properties against cognitive impairment in the elderly [27], thus making it a nootropic herb [13]. The method was further validated with blind mixtures and applied to commercial herbal products to verify labelling compliance.

## 2. Materials and Methods

### 2.1. Plant Species and Commercial Samples

Leaf and stem tissues from *B. monnieri* were kindly provided by the Botanic Garden of Helsinki (Finland) and Berlin (Germany). Plant tissues were oven-dried at 30 °C in the dark for 5 days. After drying, the plant tissue was submerged in liquid nitrogen and then ground with a mortar and pestle. In addition, 72 non-target species, including medicinal plants, herbs and spices, as well as legumes, cereals and animal species used as food, were assessed for the specificity of the designed molecular markers (Table 1). Plant and animal tissues were acquired from commercial shops and botanic gardens. Commercial herbal products labelled as Bramhi or *B. monnieri* (6 samples), Gotu Kola or *C. asiatica* (11 samples) and *G. biloba* (1 sample) were acquired at local stores and through the internet.

**Figure 1:**
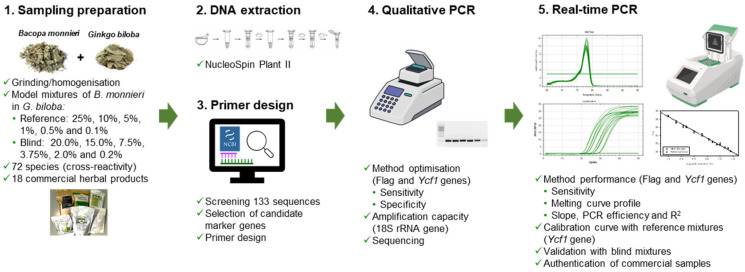
Schematic representation of the experimental workflow for real-time PCR method development targeting *B. monnieri*.

For method development, a reference mixture containing 25% *B. monnieri* dried leaves in *G. biloba* was prepared and further used for the subsequent mixtures of 10 to 0.1% by serial additions of ground *G. biloba* material. For method validation, seven blind mixtures were independently prepared as described for the reference mixtures, containing 20 to 0.2% (*w*/*w*) *B. monnieri* in *G. biloba* (Figure 1). The tissues were ground with a mortar/pestle or using a Grindomix GM200 laboratory mill (Retsch, Haan, Germany).

### 2.2. DNA Extraction

DNA extracts of plant material (50 mg), including reference and blind mixtures, commercial samples and cross-reactivity species were obtained using the NucleoSpin Plant II kit (Macherey-Nagel, Düren, Germany) with PL1 lysis buffer, according to the manufacturer’s protocol with minor adjustments as described by Biltes et al. [28]. DNA from animal tissues was extracted with the NucleoSpin Food kit (Macherey-Nagel, Düren, Germany), following the manufacturer’s instructions with minor adjustments. The extracts were stored at −20 °C until further analysis. The purity and yield of the DNA extracts were measured using UV spectrophotometry [28].

### 2.3. Screening of Molecular Markers

Firstly, a screening of 133 sequences available in the NCBI database (https://www.ncbi.nlm.nih.gov/ accessed on 31 January 2023) was performed to identify candidate markers specific to *B. monnieri*. The selection criteria were based on sequence identity: sequences showing more than 85% identity with non-target species were discarded, and the remaining sequences were evaluated for the design of specific, high-performance primers. Accordingly, the nuclear gene coding for the flavonoid glucosyltransferase (Flag) and the chloroplast gene Ycf1 photosystem I assembly protein (*Ycf1*) were selected for primer design (Table 2). In silico analysis of sequences and primers was conducted using Nucleotide BLAST (https://blast.ncbi.nlm.nih.gov/Blast.cgi, accessed on 30 May 2024) and primer-BLAST (https://www.ncbi.nlm.nih.gov/tools/primer-blast/, accessed on 30 May 2024) tools. Additionally, the absence of hairpin formation and self-hybridisation, as well as primer properties, were checked using the Oligo Calculator software v3.19 (http://oligocalc.eu/, accessed on 30 May 2024).

To verify the amplification capacity of DNA extracts, primers targeting the nuclear 18S rRNA genes were used as universal eukaryotic markers (Table 2). Primers were synthesised by Eurofins MWG Operon (Ebersberg, Germany).

### 2.4. Qualitative PCR

Qualitative PCR assays were performed in a total reaction volume of 25 μL, containing 10–20 ng of DNA, 1 U of SuperHot Taq DNA Polymerase, 1× PCR buffer (67 mM Tris-HCl, pH 8.8, 16 mM (NH_4_)_2_SO_4_, 0.01% Tween 20; Genaxxon Bioscience GmbH, Ulm, Germany), 3.0 mM of MgCl_2_ (Genaxxon Bioscience GmbH, Ulm, Germany), 200 µM of each dNTP (Grisp, Porto, Portugal), and 200 to 240 nM of each primer (Table 2). The reactions were performed in a thermal cycler MJ Mini (Bio-Rad, Hercules, CA, USA) using the following program: initial denaturation at 95 °C for 5 min, followed by 40 cycles (except for primers 18SRG-F/18SRG-R with 33 cycles, and 35 cycles for EG-F/EG-R and 18SEU-F/18SEU-R) at 95 °C for 30 s, 58 to 65 °C (according to Table 2) for 30 s and 72 °C for 30 s, with a final extension at 72 °C for 5 min. The amplified fragments were analysed by electrophoresis in a 1.5% agarose gel as described by Biltes et al. [28].

### 2.5. Sequencing

For confirmation purposes, a region of the *Ycf1* gene was partially sequenced using the primers BMYcf1S-F/BMYcf1S-R (Table 2). The reaction mix followed the same protocol as described above, containing 60 ng of *B. monnieri* DNA. The PCR fragments were purified using the GRS PCR & Gel Band Purification Kit (GRISP, Porto, Portugal) to remove any eventual interfering components and sent to a specialised facility for sequencing (Eurofins Genomics, Ebersberg, Germany). The fragment was double sequenced, which involved the direct sequencing of both strands in opposite directions to allow for production of two complementary sequences with high quality. BioEdit v7.2.5 software (Ibis Biosciences, Carlsbad, CA, USA) and FinchTV v1.4.0 software (Geospiza, Seattle, WA, USA) were used to align sequencing data and to analyse the electropherograms, respectively.

### 2.6. Real-Time PCR

For real-time PCR amplifications, the reactions were carried out in a total volume of 20 µL, containing 2 µL of DNA (10 ng or 10 ng to 1 pg for 10-fold serial dilution curve), 1× SsoFast EvaGreen Supermix (Bio-Rad Laboratories, Hercules, CA, USA) and 300 nM of BMYcf1-F/BMYcf1-R primers (Table 2). A fluorometric CFX Connect Real-Time PCR Detection System (Bio-Rad Laboratories, Hercules, CA, USA) was used to perform the amplifications under the following conditions: 95 °C for 5 min, followed by 50 cycles at 95 °C for 10 s, 62 °C for 20 s, and 72 °C for 30 s, with the fluorescent signal being collected at the end of each cycle. For melting curve analysis, the following program was used: 95 °C for 1 min, 65 °C for 3 min; melting curve ranging from 70.0 to 95.0 °C, with temperature increments of 0.2 °C/10 s. The fluorescence signal was collected at the end of each increment. Real-time PCR data and melting curve data were analysed using Bio-Rad CFX Maestro 2.3 (Bio-Rad Laboratories, Hercules, CA, USA). Samples were amplified in triplicate at least in three independent runs.

Real-time PCR calibration curves were constructed with amplified 10-fold serially diluted *B. monnieri* DNA extracts (10 ng–1 pg), which plotted the Cq values versus the log of *B. monnieri* DNA for the determination of the absolute limit of detection (LOD) and quantification (LOQ). In addition, a calibration model accounting for matrix and processing effects was constructed using the extracts of reference mixtures of *B. monnieri* in *G. biloba* (0.1–25%) amplified with an identical DNA concentration (5 ng/µL), targeting the best performing gene. Therefore, the Cq values of reference mixtures as individual calibrators versus the log of the *B. monnieri* percentage were plotted and used to estimate *B. monnieri* content in blind and commercial samples by curve interpolation. For the development and validation of the real-time PCR method, the MIQE (Minimum Information for Publication of Quantitative Real-Time PCR Experiments) guidelines [32] and the minimum performance requirements set by the European Network of GMO Laboratories were carefully considered [33]. Accordingly, acceptable PCR efficiency should fall within 90–110%, the slope in the range of −3.6 and −3.1, and the correlation coefficient (*R*^2^) ≥ 0.98. The LOD indicates the lowest amount or concentration of the analyte in a sample detectable with a 95% confidence level to ensure no more than 5% false negative results. The LOQ should be equal to or lower than the minimum amount or concentration covered by the dynamic range. The dynamic range should cover a minimum of 3–4 orders of magnitude [33].

## 3. Results

### 3.1. DNA Quality, Selection of Target Markers and Specificity

Generally, DNA extracts of plant material, including reference mixtures and commercial samples, exhibited suitable yields and purities for PCR amplification, which were in the ranges of 50.3–79.4 ng/µL and 1.6–1.7, respectively. The results of PCR assays targeting the 18S rRNA as a universal eukaryotic region demonstrated the high amplification capacity of all extracts, which is particularly relevant for commercial samples and DNA extracts for specificity testing to discard any false negatives.

In silico analysis revealed that the designed new primers targeting the Flag (BMFlagA-F/BMFlagA-R) and *Ycf1* (BMYcf1-F/BMYcf1-R) genes (Table 2) are suitable candidate markers for the specific detection of *B. monnieri*. The first is a nuclear gene, while the latter is a chloroplast gene and a multicopy region that provides higher sensitivity. The results of PCR assay optimisation targeting both gene markers demonstrate their adequacy in detecting *B. monnieri*, with superior sensitivity for the *Ycf1* gene (0.1 pg of DNA) as expected (Figure S1, Supplementary Materials).

For specificity testing, both primer pairs were assayed using 72 non-target species commonly found as ingredients of plant food supplements, herbal products and foods. The results showed that both PCR assays are specific for *B. monnieri* (Table 1).

### 3.2. Real-Time PCR Method Development

**Figure 2:**
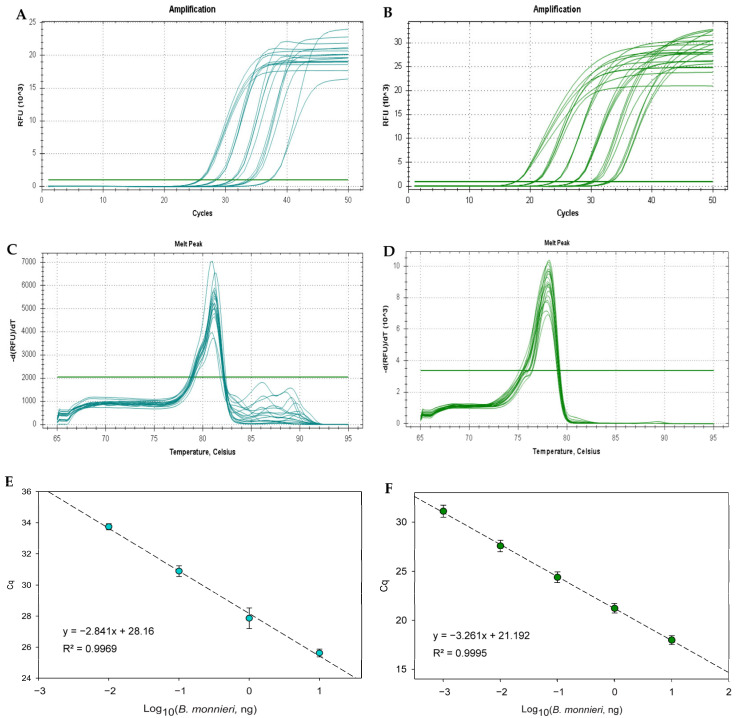
Real-time PCR amplification (**A**,**B**), melting (**C**,**D**) and calibration curves (**E**,**F**) targeting the genes coding for flavonoid glucosyltransferase (**A**,**C**,**E**) and Ycf1 photosystem I assembly protein (**B**,**D**,**F**) using a 10-fold serially diluted *B. monnieri* DNA extract ranging from 10 ng to 10 pg (*n* = 12 replicates). Legend: Green horizontal line, threshold line.

Based on the previous results, BMFlagA-F/BMFlag-R and BMYcf1-F/BMYcf1-R were considered suitable primer pairs for *B. monnieri*-specific detection. Thereafter, real-time PCR assays with EvaGreen dye were developed and their performance evaluated and compared. Figure 2 shows the amplification curves, melting peaks and respective calibration curve for both targets using a 10-fold serially diluted *B. monnieri* DNA extract (10 ng to 1 pg). While the *Ycf1* marker provided melting curves with single melt peaks at 77.90 ± 0.27 °C (Figure 2D), inferring the amplification of single PCR products, the Flag gene displayed melt peaks at 81.10 ± 0.14 °C (Figure 2C), but with slight shoulders that suggest more than one type of amplicon. This finding negatively affected the calibration curve slope (−2.841), with a consequent effect on PCR efficiency (124.9%); both parameters were out of acceptable intervals, though linearity was not compromised (*R*^2^ = 0.9969) (Figure 2E).

On the other hand, the calibration curve targeting the *Ycf1* gene exhibited acceptable parameters regarding slope (−3.261), PCR efficiency (102.6%) and coefficient correlation (*R*^2^ = 0.9995) (Figure 2F), suggesting high performance of the assay. Additionally, it reached a higher sensitivity than the Flag gene with an absolute LOD of 1 pg of bacopa DNA, as it was the lowest level that amplified all the replicates (*n* = 12) (Table S1, supplementary data). The absolute LOQ was established as equal to the absolute LOD since it was within the linear range. The dynamic range covered four orders of magnitude, which is according to the recommended for this type of assay.

**Figure 3:**
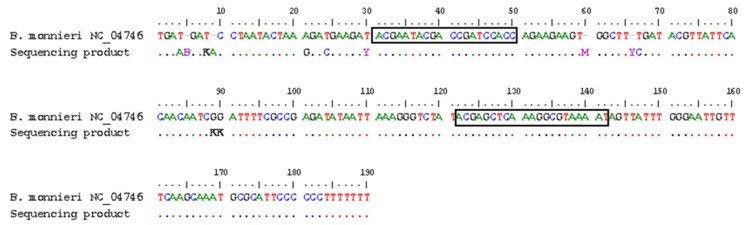
Alignment of sequences of NCBI database (NCBI accession no. NC_04746) and the amplicon of *B. monnieri* specimen obtained using BMYcf1S-F/BMYcf1S-R primers targeting the gene coding for the Ycf1 photosystem I assembly protein. The black text boxes represent the BMYcf1-F and BMYcf1-R primers, respectively.

To confirm the sequence identity of the *Ycf1* fragment, new primers were designed to amplify a 370 bp amplicon encompassing the target region of 110 bp, thus enabling its reliable sequencing. The alignment of the amplicon with the sequence retrieved from the NCBI revealed an almost full homology of sequences, confirming the target fragment (Figure 3). The specificity of the target *Ycf1* sequence was further complemented following sequence alignment of *B. monnieri* with all the available sequence species of the same family retrieved from the NCBI. The alignment results are displayed in Figure S2 (Supplementary Material), showing the substantial differences among sequences, which prevent their amplification with the designed new primers.

**Figure 4:**
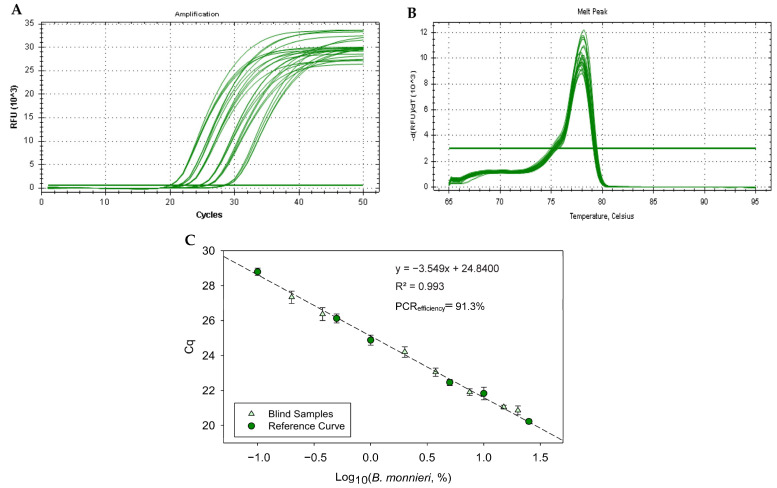
Real-time PCR amplification (**A**), melting (**B**) and calibration curves (**C**) targeting the gene coding for Ycf1 photosystem I assembly protein using primers BMYcf1-F/BMYcf1-R and DNA extracts of reference mixtures of dried *B. monnieri* leaves in *G. biloba*. Legend: (●) reference mixtures (25.0% 10.0%, 5.0%, 1.0%, 0.5% and 0.1%), (▲) blind samples (20.0%, 15.0%, 7.5%, 3.75%, 2.0%, 0.375% and 0.2%) (*n* = 3 replicates, 3 independent runs).

Considering the optimum performance of the real-time PCR assay targeting the *Ycf1* gene, a quantitative model was developed using the reference mixtures of dried *B. monnieri* leaves in *G. biloba* (25%, 10%, 5%, 1%, 0.5% and 0.1%). Figure 4 shows the amplification curves, melting peaks and respective calibration curve. The results of melting curve analysis suggest the amplification of single amplicons with melting temperatures of 77.96 ± 0.12 °C (Figure 4B), which agrees with previous findings using serially diluted bacopa DNA (Figure 2D). The obtained calibration curve (Figure 4C) exhibited high performance as inferred from the parameters of slope (−3.549), PCR efficiency (91.3%) and *R*^2^ (0.993), with a dynamic range covering six reference mixtures and three orders of magnitude, which were all within the acceptance criteria. The assay achieved LOD and LOQ values of 0.1%, as all the 12 replicates of 3 independent assays amplified within the linear range.

### 3.3. Validation of Method

To validate the method, seven blind mixtures of *B. monnieri* in *G. biloba* (20.0%, 15.0%, 7.50%, 3.75%, 2.0%, 0.375% and 0.20% (*w*/*w*)) were quantified using the real-time PCR calibration curve targeting the *Ycf1* gene (Figure 4C). The comparison of actual and estimated bacopa contents, as well as precision and accuracy data, are summarised in Table 3. The precision was expressed by the coefficient of variation (CV), representing the relative standard deviation of estimates under repeatability conditions. CV data varied between 7.0 and 24.6% and were within the acceptance criteria (≤25%) [33], with the highest value attributed to the lowest tested concentration level (0.20%), where less reliability is normally expected. The trueness of the assay was expressed by the bias or error, demonstrating the closeness of agreement between the actual and estimated bacopa contents. The calculated bias values were between −11.4 and 23.8%, which complied with the acceptable range of ±25% [33].

### 3.4. Authentication of Commercial Herbal Products

The applicability of the proposed real-time PCR method was then assessed by analysing commercial herbal products. The *B. monnieri* contents were determined interpolating the calibration curve of Figure 4C. The samples included products labelled with bacopa or Brahmi (6), products labelled as Gotu kola or *C. asiatica* (11) and one sample without any of the two species (*G. biloba*) (Table 4). Of the six samples labelled with bacopa/Brahmi, the presence of *B. monnieri* was confirmed in only three*;* two of them (P1 and P2) had estimates above the quantitative range and thus likely containing 100% content, while in one (P5), the content (4.19%) was much smaller than the labelled content (100% Brahmi). Concerning the samples labelled with *C. asiatica*, none of them was positive to *B. monnieri* as would be anticipated. As expected, the ginkgo-containing sample (P18) was negative to *B. monnieri*. It is important to highlight that all the samples tested positively for the 18S rRNA gene marker using the EG-F/EG-R primers (Table 4), thus discarding any false negative results. Therefore, out of the 6 samples labelled with bacopa or Brahmi, 4 suggest (P3 to P6) adulteration practices by the absence of or substantial reduction in *B. monnieri*. All the other non-bacopa declaring samples did not indicate any adulteration by addition of *B. monnieri*.

Moreover, following the development of a new real-time PCR method to detect and quantify *C. asiatica* in herbal products, we were able to complement results for some samples regarding this species [28]. Accordingly, the five samples labelled as 100% *C. asiatica* (P8, P12–P14, P17) contained high amounts of *C. asiatica* (>25%), while in those labelled as containing between 10 and 30% *C. asiatica* (P7, P9–P11), the species was not detected, except for P16, which was estimated to contain 17% *C. asiatica* [28]. These data further suggest the absence of both *B. monnieri* and *C. asiatica* in samples (P7, P9–P11), while others (P8, P12–P14, P16, P17) complied with the labelled amount of *C. asiatica*.

## 4. Discussion

In the present work, a real-time PCR-based method was developed to establish a true quantitative model of *B. monnieri* in herbal material. For this purpose, a reference curve was constructed using binary mixtures with known concentrations of the target plant mixed in *G. biloba*. *G. biloba* was selected due to its popularity and known nootropic effects [13,27]. Given its widespread use as one of the most popular medicinal plants globally, it is frequently included as a co-ingredient in neurological herbal formulations. Using *G. biloba* as a standardised and controlled matrix for method validation ensures reproducibility and reliable quantification of *B. monnieri* in complex herbal products. The developed method, based on matrix-adapted standards, accounted for the matrix effect since all the standards were individual plant extracts [34], not serially diluted DNA. Despite that, a calibration curve within 25–0.1% bacopa in ginkgo plant material was set, showing acceptable performance parameters of slope, linearity, PCR efficiency and dynamic range coverage. To validate the method, the precision and accuracy were assessed based on seven blind mixtures. According to Kang [34], for food analysis using real-time PCR methods, bias and CV are generally considered acceptable within a maximum range of 25–30%. The herein proposed method based on matrix-adapted standards exhibited CV and bias data below 25%, which confirms its precision and trueness to estimate bacopa in herbal products. However, the application of this approach to other plant matrices might affect the target species amplification due to matrix effects and the presence of inhibitor compounds [28,35]. This can be noted during real-time PCR amplification, when observing fallen curve profiles. To overcome this problem, different DNA extraction protocols should be attempted, and/or a new calibration curve should be prepared, simulating as closely as possible the plant matrix to be evaluated.

To advance a real-time PCR method suitable for authenticating plants or commercial products, parameters such as specificity, sensitivity, PCR efficiency, linearity and dynamic range must be addressed and aligned with the specified criteria outlined in the international guidelines or standards [32,33,34]. In this regard, to guarantee the specificity of the method, a nuclear and a chloroplast gene marker were selected among several sequences in the NCBI and scrutinised by in silico and in vitro approaches using 72 non-target relevant species as ingredients normally used in herbal products and/or food supplements [36]. Both the Flag and *Ycf1* gene markers were revealed to be specific, as confirmed by the in silico screening results and by the absence of any PCR amplicon besides the target plant species. The Flag gene was selected as a nuclear marker coding for the flavonoid glucosyltransferase, while *Ycf1* is a chloroplast gene considered as a promising barcode marker for land plants [37]. Thereafter, both markers were assessed by real-time PCR with EvaGreen dye as a more economic and simple approach than a probe system. Melting curve analysis confirmed fragment specificity for the *Ycf1* gene marker, while Flag exhibited neither single melt peaks nor adequate real-time PCR kinetics. The amplicon identity was further confirmed by sequencing analysis, though with minor variability, which was anticipated, as this is a multicopy region [38]. The developed real-time PCR assay targeting the *Ycf1* gene complied with the minimum performance requirements regarding the slope of the standard curve, linearity, PCR efficiency and dynamic range for the detection and quantification of *B. monnieri* DNA, achieving an absolute LOD and LOQ of 1 pg of bacopa DNA. This sensitivity was similar to the one obtained by Xu et al. [18], who targeted a multicopy region (ITS2) by species-specific PCR and real-time PCR with SYBR Green dye. However, the referred authors neither provided a calibration curve nor any performance parameter data to further compare the results.

The application of the developed real-time PCR method to commercial herbal products suggests an important level of adulteration due to full or partial substitution of *B. monnieri* with other plant(s) in four out of six Brahmi/bacopa products. Nonetheless, the potential addition of *B. monnieri* to *C. asiatica* products, which can also be called Brahmi, was not detected in any of the 11 tested products. This finding agrees with the high level of adulteration (55.6%) identified in Brahmi products by DNA metabarcoding [16]. DNA metabarcoding is a high-throughput sequencing approach that allows for the simultaneous detection of multiple taxa, including *B. monnieri* and *C. asiatica*. Despite the high sensitivity of high-throughput sequencing, it is a rather qualitative approach as it relies on the percentage of reads to estimate plant species, which is affected by several factors, such as the quality and integrity of extracted DNA, the library preparation method in PCR and sequencing platform [16]. Additionally, DNA metabarcoding requires bioinformatic experts and much more costly equipment and consumables than real-time PCR. Therefore, the herein-developed method allows for the quantification of *B. monnieri* in herbal products, and it is one of the most comprehensive and fully validated approaches available so far. Keeping in mind the current presence of *C. asiatica* in herbal products and the high level of adulteration detected in the analysed bacopa/Brahmi products, we have already advanced the development of a real-time PCR method to detect and quantify this species, which further complements the results of some samples, suggesting that those declared to be 100% *C. asiatica* comply with the declared species, but those with lower *C. asiatica* proportions mostly indicate the absence of the species [28].

The development of a novel real-time PCR-based tool to authenticate *B. monnieri* commercial products represents a new achievement in the field. To our knowledge, such a tool has not been previously developed. As highlighted earlier, the Brahmi market is expanding, with various herbal products being globally commercialised or developed in different forms and dosages containing this nootropic plant. This increases the risk of plant substitution or addition of illegal substances, leading to adulteration practices. In response to these challenges, a robust, accurate, sensitive and reproducible real-time PCR method was developed to address these concerns.

## 5. Conclusions

In the present work, a novel molecular marker was proposed for the species-specific detection of *B. monnieri* by qualitative PCR and real-time PCR. To our knowledge, the developed real-time PCR method is one of the most comprehensive and fully validated approaches available so far to detect/quantify *B. monnieri*, demonstrating high specificity and sensitivity for the target species, down to 1 pg of bacopa DNA. Additionally, a calibration model was proposed using reference mixtures as matrix-adapted standards of *B. monnieri* in *G. biloba.* The method exhibited high analytical performance, meeting the acceptance criteria regarding calibration curve slope, PCR efficiency and coefficient of correlation amd covering a linear dynamic range of 25–0.1% (*w*/*w*) of *B. monnieri* in *G. biloba*. Validation of the quantitative real-time PCR method with blind mixtures highlights the high precision and accuracy of the proposed method to estimate bacopa in herbal products. Its applicability was further demonstrated by analysing commercial herbal products, which revealed an important level of adulteration in bacopa/Brahmi-labelled products (60%) by the absence of or reduction in bacopa. This study proposes a comprehensive and validated quantitative real-time PCR method for the botanical authentication of *B. monnieri* in herbal products as a powerful tool for control laboratories and regulatory authorities to ensure labelling compliance.

## Figures and Tables

**Table 1:** Animal and plant species used in specificity testing using primers targeting the genes coding for the flavonoid glucosyltransferase (Flag) and Ycf1 photosystem I assembly protein (*Ycf1*) of *B. monnieri*.

Common Name	Species	Qualitative PCR ^a^		
		18S rRNA	Flag	*Ycf1*
Bacopa	*Bacopa monnieri* ^b^	+++	+++	+++
Bacopa	*B. monnieri* ^c^	+++	+++	+++
House cricket	*Acheta domestica*	+++	-	-
Onion	*Allium cepa*	+++	-	-
Garlic	*Allium sativum*	+++	-	-
Lemon verbena	*Aloysia citrodora*	+++	-	-
Ananas	*Ananas comosus*	+++	-	-
Daisy-leaved toadflax	*Anarrhinum bellidifolium*	+++	-	-
Carniolan honeybee	*Apis mellifera carnica*	+++	-	-
Celery	*Apium graveolens*	+++	-	-
Common bearberry	*Arctostaphylos uva-ursi*	+++	-	-
Argan	*Argania spinosa* L.	+++	-	-
Spirulina	*Arthrospira platensis*	+++	-	-
Cow	*Bos taurus*	+++	-	-
Green tea	*Camellia sinensis*	+++	-	-
Spanish chestnut	*Castanea sativa*	+++	-	-
Gotu kola	*Centella asiatia*	+++	-	-
Bitter orange	*Citrus aurantium*	+++	-	-
Borututu	*Cochlospermum angolense*	+++	-	-
Coriander	*Coriandrum sativum*	+++	-	-
Oysters	*Crassostrea angulata*	+++	-	-
Hawthorn	*Crataegus monogyna*	+++	-	-
Turmeric	*Curcuma longa*	+++	-	-
Lemongrass	*Cymbopogon citratus*	+++	-	-
Artichoke	*Cynara cardunculus* var*. scolymus*	+++	-	-
Scotch broom	*Cytisus scoparius*	+++	-	-
Truncate donax	*Donax trunculus*	+++	-	-
Siberian ginseng	*Eleutherococcus senticosus*	+++	-	-
Common horsetail	*Equisetum arvense*	+++	-	-
Messmate stringybark	*Eucalyptus obliqua* L’Hér	+++	-	-
Common fennel	*Foeniculum vulgare*	+++	-	-
Cod	*Gadus morhua*	+++	-	-
Chicken	*Gallus gallus domesticus*	+++	-	-
Herb-robert	*Geranium robertianium*	+++	-	-
Ginkgo	*Ginkgo biloba*	+++	-	-
Soybean	*Glycine max* L.	+++	-	-
St. John’s wort	*Hypericum perforatum*	+++	-	-
Laurel	*Laurus nobilis*	+++	-	-
White leg shrimp	*Litopenaeus vannamei*	+++	-	-
Chamomile	*Matricaria chamomilla*	+++	-	-
Lemon balm	*Melissa officinalis*	+++	-	-
Peppermint	*Mentha piperita* L.	+++	-	-
Common ling	*Molva molva*	+++	-	-
Incense	*Pittosporum undulatum* Vent.	+++	-	-
Olive	*Olea europaea* L.	+++	-	-
Domestic sheep	*Ovis aries*	+++	-	-
Asian ginseng	*Panax ginseng*	+++	-	-
Passion fruit	*Passiflora edulis*	+++	-	-
Avocado	*Persea americana*	+++	-	-
Parsley	*Petroselinum crispum*	+++	-	-
Forkbeard	*Phycis phycis*	+++	-	-
Stone pine	*Pinus pinea*	+++	-	-
Common plum	*Prunus domestica*	+++	-	-
Guava	*Psidium guajava*	+++	-	-
Sea radish	*Raphanus raphanistrum* subsp*. maritimus*	+++	-	-
Blackberry	*Rubus fruticosus* L.	+++	-	-
Sage	*Salvia officinalis*	+++	-	-
Rosemary	*Salvia rosmarinus*	+++	-	-
Atlantic chub mackerel	*Scomber colias*	+++	-	-
Egyptian senna	*Senna alexandrina*	+++	-	-
Common cuttlefish	*Sepia officinalis*	+++	-	-
White mustard	*Sinapis alba*	+++	-	-
Tomato	*Solanum lycopersicum*	+++	-	-
Arizona necklacepod	*Sophora arizonica*	+++	-	-
Common dandelion	*Taraxacum officinale*	+++	-	-
Yellow mealworm	*Tenebrio molitor*	+++	-	-
White garden snail	*Theba pisana*	+++	-	-
European white lime	*Tilia tomentosa*	+++	-	-
Hare’s-foot clover	*Trifolium arvense*	+++	-	-
Trefoil	*Trifolium* sp.	+++	-	-
Broad bean	*Vicia faba*	+++	-	-
Grape vine	*Vitis vinífera* L.	+++	-	-
Maize	*Zea mays*	+++	-	-
Ginger	*Zingiber officinale*	+++	-	-

^a^ +++, strong PCR fragment; -, no PCR fragment. ^b^ Origin: Botanic Garden of Helsinki; ^c^ Origin: Botanic Garden of Berlin.

**Table 2:** Primer sequences targeting *B. monnieri* gene markers and a conserved eukaryotic region, respective PCR products and PCR conditions.

Target Gene	Primers	Sequence (5→3)	Amplicon	Annealing Temperature	Concentration (nM)	NCBI Accession/ Reference
Flavonoid glucosyltransferase	BMflagA-F BMflagA-R	CGATTAAGGTTGTCGCTGCG TATCCCTGTTCCAGCTCCTCA	100 bp	58 °C	200	FJ586246.1
Ycf1 photosystem I assembly protein	BMYcf1-F BMYcf1-R	ACGAATACGACCGATCCACC ATTTTACGCCTTTGAGCTCGT	110 bp	62 °C	200	NC_047469.1
	BMYcf1S-F BMYcf1S-R	ATCAGGAGAACGTCAAGAAGATGTA TGATTCTTTTGTCCCTACCCAATTTTG	370 bp	60 °C	200	NC_047469.1
Nuclear 18S rRNA	18SRG-F 18SRG-R	CTGCCCTATCAACTTTCGATGGTA TTGGATGTGGTAGCCGTTTCTCA	113 bp	65 °C	240	[29]
	EG-F EG-R	TCGATGGTAGGATAGTGGCCTACT TGCTGCCTTCCTTGGATGTGGTA	109 bp	63 °C	240	[30]
	18SEU-F 18SEU-R	TCTGCCCTATCAACTTTCGATGG TAATTTGCGCGCCTGCTG	140 bp	60 °C	240	[31]

**Table 3:** Validation data using quantitative real-time PCR applied to blind samples of *B. monnieri* in *G. biloba* using BMYcf1-F/BMYcf1-R primers.

Sample	Cq ^a^ (Mean ± SD)	*B. monnieri* (% *w*/*w*)		SD	CV ^b^ (%)	Bias ^c^ (%)
		Actual	Mean Predicted			
A	20.92 ± 0.32	20.0	17.7	2.10	11.8	−11.4
B	21.09 ± 0.15	15.0	14.3	1.00	7.0	−4.7
C	21.92 ± 0.26	7.5	8.19	1.00	12.2	9.2
D	23.06 ± 0.23	3.75	3.87	0.59	15.3	3.2
E	24.17 ± 0.31	2.0	1.83	0.32	17.5	−8.4
F	27.09 ± 0.58	0.375	0.45	0.08	18.8	19.3
G	26.37 ± 0.37	0.20	0.25	0.06	24.6	23.8

^a^ Mean cycle of quantification (Cq) values ± standard deviation (SD) (*n* = 4) of three independent assays. ^b^ Coefficient of variation (CV). ^c^ Bias = ((mean estimated value—real value)/real value) × 100.

**Table 4:** Application of the quantitative real-time PCR assay using BMYcf1-F/BMYcf1-R primers for the detection and quantification of *B. monnieri* in commercial samples.

Sample	Origin	Relevant Label Information	Qualitative PCR ^a^		Real-Time PCR	
			EG-F/ EG-R	BMYcf1-F/ BMYcf1-R	BMYcf1-F/BMYcf1-R (Cq ± SD) ^b^	Estimated Content (*w*/*w*, %) (Mean ± SD) ^c^
P1	Germany	100% Organically grown Bacopa powder	+	+	18.72 ± 0.07	>25%
P2	India	100% *B. monnieri* leaf powder	+	+	19.95 ± 0.01	>25%
P3	India	100% *B. monnieri* powder	+	+	31.64 ± 0.15	<LOD
P4	India	100% Bacopa leaf powder	+	−	ND ^d^	
P5	India	100% Brahmi	+	+	22.94 ± 0.09	4.19 ± 0.22
P6	India	100% Brahmi whole plant powder	+	−	ND	
P7	EU	25% Gotu kola (*Hydrocotyle asiatica*)	+	−	ND	
P8	Portugal	100% *Centella asiatica* leaves	+	−	ND	
P9	India	30% Gotu Kola (*C. asiatica*)	+	−	ND	
P10	Portugal	10% *C. asiatica* aerial parts	+	−	ND	
P11	Portugal	15% *C. asiatica* aerial parts	+	−	ND	
P12	Portugal	100% *C. asiatica* leaves	+	−	ND	
P13	India	100% *C. asiatica* leaves	+	−	ND	
P14	Portugal	100% *C. asiatica* leaves	+	−	ND	
P15	India	10% *C. asiatica* leaves	+	−	ND	
P16	India	6.76% Brahmi (*C. asiatica*) leaves	+	−	ND	
P17	Portugal	100% *C. asiatica* leaves	+	−	ND	
P18	Portugal	15% *Ginkgo biloba* leaves	+	−	ND	

^a^ (+) Positive amplification; (−) Negative amplification. ^b^ Mean cycle of quantification (Cq) values ± standard deviation (SD) (*n* = 4 replicates). ^c^ Mean content (%) values ± SD (*n* = 4). ^d^ ND, not detected.

## Data Availability

The original contributions presented in this study are included in the article/Supplementary Material. Further inquiries can be directed to the corresponding author.

## Supplementary Materials

The following supporting information can be downloaded at https://www.mdpi.com/article/10.3390/foods14183275/s1. Table S1: Amplification and calibration curve data of real-time PCR assays targeting the genes coding for the flavonoid glucosyltransferase (Flag) and Ycf1 photosystem I assembly protein (*Ycf1*) using a 10-fold serially diluted *B. monnieri* DNA extract; Figure S1: Agarose gel electrophoresis of PCR products targeting the flavonoid glucosyltransferase (A) and Ycf1 photosystem I assembly protein (B) genes using 10-fold serially diluted *B. monnieri* DNA amplified with different annealing temperatures. Legend: M, 100 bp DNA Ladder; lane 1, 10 ng; lane 2, 1 ng; lane 3, 0.1 ng; lane 4, 0.01 ng; lane 5, 1 pg; lane 6, 0.1 pg; NC, negative control. Figure S2: Alignment of NCBI blast sequences from Plantaginaceae (actual family for *B. monnieri*) and Scrophulariaceae (former family for *B. monnieri*) families showing nucleotide differences in the amplicon region of BMYcf1-F/BMYcf1-R between *Bacopa monnieri* and related species.

## Abbreviations

The following abbreviations are used in this manuscript:

CAGR	Compound annual growth rate
Cq	Cycle of quantification
CV	Coefficient of variation
EU	European Union
FDA	Food and Drug Administration
Flag	Flavonoid glucosyltransferase gene
GMO	Genetically modified organisms
HRM	High resolution melting
LOD	Limit of detection
LOQ	Limit of quantification
MIQE	Minimum information for publication of quantitative real-time PCR experiments
PCR	Polymerase chain reaction
RAPD	Randomly amplified polymorphic DNA
SCAR	Sequence-characterised amplified region
SD	Standard deviation
*Ycf1*	Ycf1 photosystem I assembly protein gene
