# Supplementary material for: Exploiting Marker Genes for Reliable Botanical Authentication of Bacopa monnieri Products
Source: Foods. 2025 Sep 21;14(18):3275. doi: 10.3390/foods14183275 (PMC12469326; doi:10.3390/foods14183275)
Supplement: Supplementary file 1 [file foods-14-03275-s001.zip › foods-3822280-supplementary.pdf]

Exploiting Marker Genes for Reliable Botanical Authentication of *Bacopa monnieri* Products

Rita Biltes, Caterina Villa, Joana Costa and Isabel Mafra *

REQUIMTE/LAQV, Faculdade de Farmácia, Universidade do Porto, Rua de Jorge Viterbo Ferreira, 228, 4050-313 Porto, Portugal

Supplementary material

Table S1. Amplification and calibration curve data of real-time PCR assays targeting the genes coding for the flavonoid glucosyltransferase (Flag) and Ycf1 photosystem I assembly protein (*Ycf1*) using a 10-fold serially diluted *B. monnieri* DNA extract.

B. monnieri DNA (ng)	Flag		Ycf1b	
	Cq ± SD[a]	Replicates (positive/total)	Cq ± SD[a]	Replicates (positive/total)
10	25.63 ± 0.25	(8/8)	17.98 ± 0.45	(8/8)
1	27.86 ± 0.66	(8/8)	21.22 ± 0.48	(8/8)
0.1	30.90 ± 0.35	(8/8)	24.39 ± 0.56	(8/8)
0.01	33.74 ± 0.19	(8/8)	27.57 ± 0.59	(8/8)
0.001	36.90 ± 0.39	(4/8)	31.11 ± 0.61	(8/8)
0.0001	ND	-	33.17 ± 0.17	(6/8)
Correlation coefficient (R^2)	0.997		0.999	
Slope	-2.841		-3.261	
Intercept	28.167		21.193	
PCR efficiency (%)	124.9		102.6	

[a] Mean cycle of quantification (Cq) values ± standard deviation (SD).

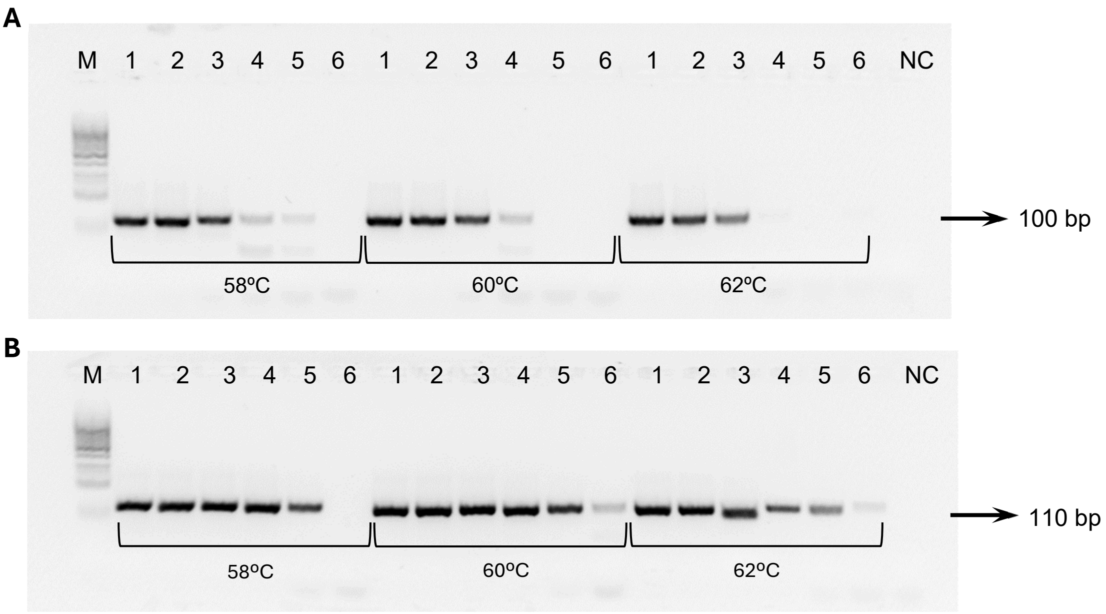

Figure S1. Agarose gel electrophoresis of PCR products targeting the flavonoid glucosyltransferase (A) and Ycf1 photosystem I assembly protein (B) genes using 10-fold serially diluted *B. monnieri* DNA amplified with different annealing temperatures. Legend: M, 100 bp DNA Ladder; lane 1, 10 ng; lane 2, 1 ng; lane 3, 0.1 ng; lane 4, 0.01 ng; lane 5, 1 pg; lane 6, 0.1 pg; NC, negative control.

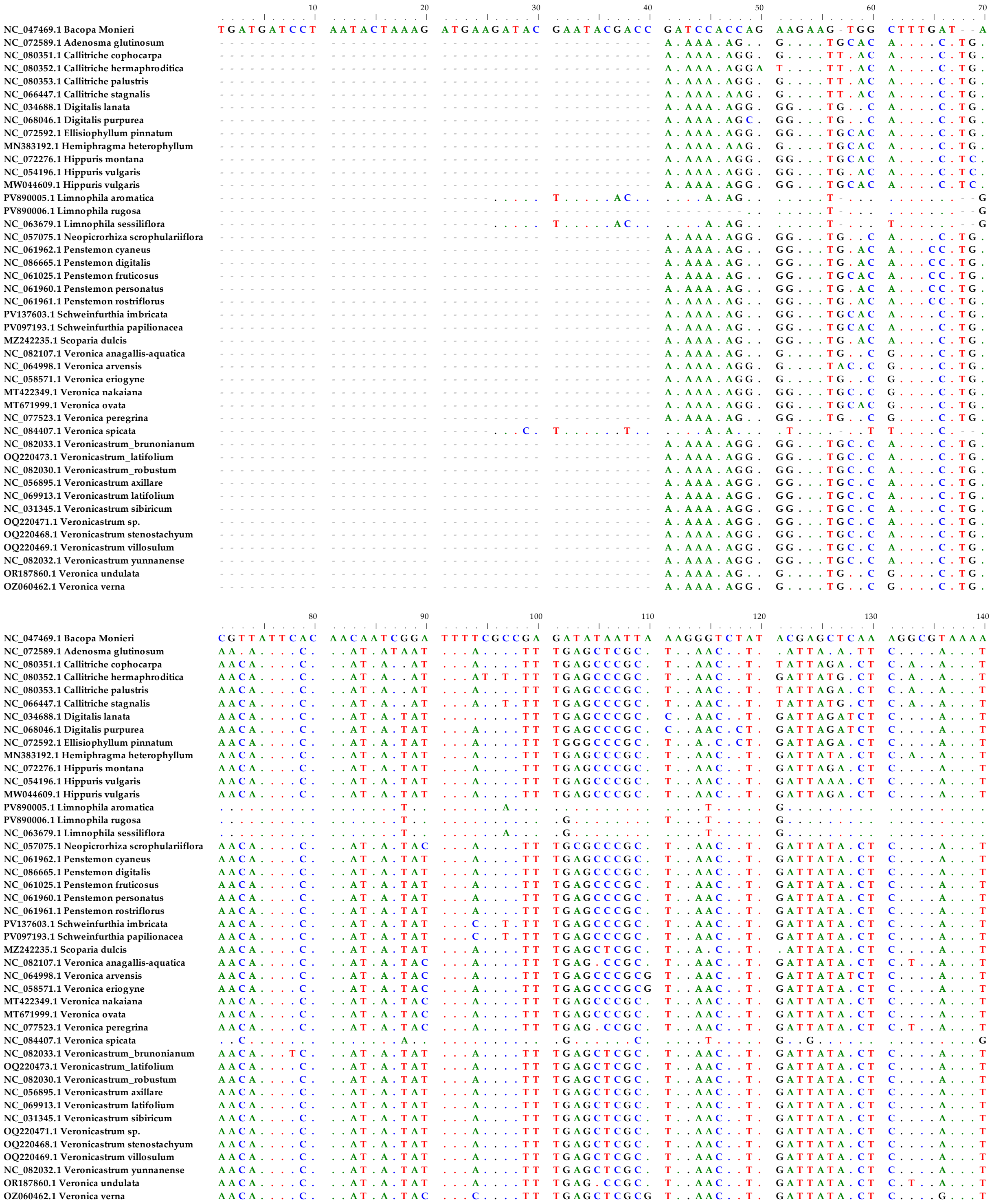

Figure S2. Alignment of NCBI blast sequences from Plantaginaceae (actual family for *B. monnieri*) and Scrophulariaceae (former family for *B. monnieri*) families showing nucleotide differences in the amplicon region of BMYcf1-F/BMYcf1-R between *Bacopa monnieri* and related species.

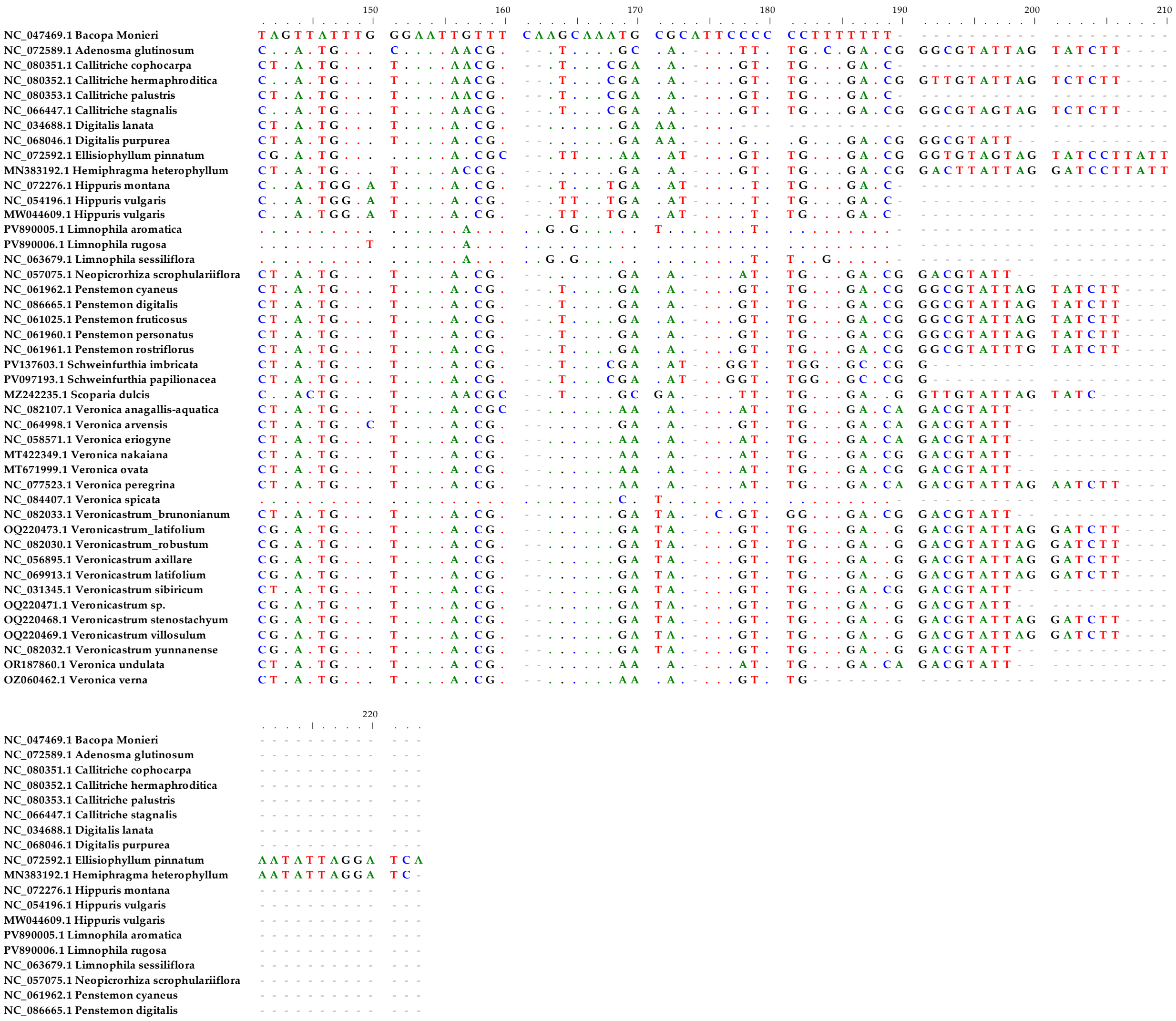

Figure S2. (Continued)
